# Using Alternative Definitions of Controls to Increase Statistical Power in GWAS

**DOI:** 10.21203/rs.3.rs-3858178/v1

**Published:** 2024-01-31

**Authors:** Sarah E. Benstock, Katherine Weaver, John Hettema, Brad Verhulst

## Abstract

Genome-wide association studies (GWAS) are underpowered due to small effect sizes of single nucleotide polymorphisms (SNPs) on phenotypes and extreme multiple testing thresholds. The most common approach for increasing statistical power is to increase sample size. We propose an alternative strategy of redefining case-control outcomes into ordinal case-subthreshold-asymptomatic variables. While maintaining the clinical case threshold, we subdivide controls into two groups: individuals who are symptomatic but do not meet the clinical criteria for diagnosis (subthreshold) and individuals who are effectively asymptomatic. We conducted a simulation study to examine the impact of effect size, minor allele frequency, population prevalence, and the prevalence of the subthreshold group on statistical power to detect genetic associations in three scenarios: a standard case-control, an ordinal, and a case-asymptomatic control analysis. Our results suggest the ordinal model consistently provides the most statistical power while the case-control model the least. Power in the case-asymptomatic control model reflects the case-control or ordinal model depending on the population prevalence and size of the subthreshold category. We then analyzed a major depression phenotype from the UK Biobank to corroborate our simulation results. Overall, the ordinal model improves statistical power in GWAS consistent with increasing the sample size by approximately 10%.

## 1 Introduction

Genome-wide association studies (GWAS) are the principal method for estimating associations between common single nucleotide polymorphisms (SNPs) and complex genetic traits such as psychiatric disorders. Over the past 20 years, more than 157,000 genome-wide significant genetic associations have been published for an extremely wide range of phenotypes (Buniello et al., 2019). These results overwhelmingly demonstrate that individual common genetic variants have extremely small effects on complex behaviors (Visscher et al., 2021; Wray et al., 2013). Because GWAS conduct millions of regression analyses, multiple testing corrections are essential to control the false positive rate (Dudbridge & Koeleman, 2004). The combination of small effect sizes and extremely high multiple testing rates emphasizes the importance of increasing statistical power of GWAS. Several techniques are used to increase the likelihood of detecting significant genetic associations. The first, and perhaps most prominent, method of increasing statistical power is increasing sample size. Many current GWAS use more than 500,000 observations (Howard et al., 2019; Levey et al., 2021; Liu et al., 2019; Okbay et al., 2022). While increasing sample size increases power, there are limitations on how many more individuals can be recruited in the future. In addition, phenotype validity is often compromised for sample size (Cai et al., 2020; Craddock et al., 2008; Flint, 1996). The second strategy is to use more detailed assessment methods that decrease the random noise in the phenotype. This deep phenotyping strategy practically translates into more accurate assessment of fewer traits potentially necessitating additional data collection (Kendler et al., 2019; Yehia & Eng, 2019). Even deeply-phenotyped studies with smaller sample sizes could still benefit from all possible increases in statistical power. Our goal is to use existing samples and, keeping within a disease model framework, disaggregate the control group into subthreshold and asymptomatic individuals. The resulting increase in phenotypic information potentially improves the power to detect more genetic associations without collecting additional data or recontacting the participants to obtain more detailed assessments.

Behavioral and psychological disorders are primarily inferred from observable symptoms or clinical judgments as opposed to objective physiological measures. For example, psychiatric disorders and symptoms are often assessed using binary or ordinal responses that may include subjective interpretations, uncertainty, or measurement error. Alternatively, it is possible to aggregate the number and severity of observed symptoms into a continuous score. While continuous data methods have more statistical power than ordinal methods, which in turn are more powerful than binary methods, symptom scores or dimensional psychopathology phenotypes may not reflect clinically relevant phenomena, as they may ignore key factors such as necessary or cardinal symptoms, perceived distress, temporal duration, or symptom clustering (Verhulst & Neale, 2021; Yang et al., 2010; Zimmerman et al., 2018; Fried & Nesse, 2015). We focus on case-control studies, as they remain the predominant approach for GWAS analyses of disease outcomes.

Case-control phenotypes tend to fall into two categories. The standard case-control model defines controls as anyone who does not meet the case criteria for the phenotype of interest. This method is easy to implement and is unbiased in large samples. The second method uses a screened control group, which is a group that, on average, has lower genetic liability for the disease outcome than a control group randomly drawn from the underlying population of asymptomatic individuals (Schork et al., 2019). An example of an extreme control group is a 'supernormal' control group which excludes individuals who do not meet the case threshold as well as those who express disorders that are frequently comorbid or highly correlated with the target phenotype. Because exclusion criteria asymmetrically removes controls but not cases, using 'supernormal' controls can result in biased estimates of the genetic associations as the cases will be enriched for all the disorders that were screened out of the control group (Kendler et al., 2019). This has the potential to bias both genetic associations and correlations. Another extreme control phenotype employs asymptomatic controls, where individuals have very minimal symptoms of the target phenotype. Using asymptomatic controls increases statistical power to detect genetic associations without inflating Type I Error rates (Gorla et al., 2023; Nudel et al., 2020). Asymptomatic controls, however, inflate effect size estimates (as a portion of the liability distribution is excluded from the analyses) which may bias post-GWAs analyses like heritability estimates (Nudel et al., 2020; Schork et al., 2019; Yap et al., 2018). Transformations that account for ascertainment of extreme controls and lifetime risk for the phenotype potentially address this bias (Schork et al., 2019; Yap et al., 2018). If the biases are appropriately addressed, the increase in statistical power when using asymptomatic controls should not be overlooked (Johnson & Abecasis, 2017).

A limited number of studies have used an alternative approach to categorizing controls to theoretically increase statistical power without biasing estimates of the genetic associations, SNP-heritability, or genetic correlations, by distinguishing symptomatic individuals who do not reach a clinical threshold for diagnosis from those who are virtually asymptomatic (Hettema et al., 2020; Otowa et al., 2016). This ordinal approach provides more information than binary diagnostic categories while maintaining the clinical validity of the cases. Importantly, the interpretation of genetic associations with the underlying genetic liability to the disorder remains than same for the standard case-control and the case-subthreshold-asymptomatic approaches (van der Sluis et al., 2013; Bienvenu et al., 1998; Kendler et al., 2013; Kendler & Gardner, 1998).

Our overarching hypothesis is that refining the definition of controls will increase the power to detect genetic associations relative to standard case-control analyses. We predict that, as the proportion of observations in the subthreshold category increases, the relative power of the ordinal model over the standard case-control model will increase due to the increase in information. Similarly, we expect case-asymptomatic control GWAS analyses to have more statistical power because excluding the subthreshold individuals will increase the effect size (genotype differences) of the association. However, if the subthreshold group gets too large, the reduction in sample size will overwhelm the increase in effect size, resulting in reductions in power. Any increases in the ability to detect genetic associations must be examined in light of population prevalence (Hong & Park, 2012) and minor allele frequencies (MAF), two factors which are known to affect statistical power (Sham & Purcell, 2014). We tested these hypotheses using a simulation study that compares the relative power of the three approaches: standard case-control, case-asymptomatic controls, and an ordinal case/subthreshold/asymptomatic phenotype. We then conducted a real data demonstration analysis where we applied this coding scheme to a major depression phenotype in the UK Biobank.

## 2 Methods

### 2.1 | Simulation Analyses

We conducted a series of simulation analyses to examine differences in the ability to detect genetic associations depending on the operationalization of the control group in case-control analyses. Specifically, we subdivided the control group into two categories following the liability threshold model. The “subthreshold” group consisted of observations that, while symptomatic, do not reach a clinical threshold for diagnosis, and thus, were not sufficiently severe to be considered “cases” but nevertheless, were relatively close to the case threshold. The “asymptomatic” control group consisted of observations that were sufficiently unaffected to unambiguously be considered controls. This resulted in three definitions of the control group: standard controls that include both subthreshold and asymptomatic controls; an ordinal control group, where subthreshold controls are treated as an intermediate ordinal category; and an asymptomatic control group, where the subthreshold controls are excluded from the analysis.

All data were simulated and analyzed in R (R Core Team, 2021). SNP data was simulated to ensure that everything remained within Hardy-Weinberg equilibrium. Phenotypic data was simulated using a quantile distribution that varied on the defined population prevalence of the outcome and the prevalence of the subthreshold group within the population. Three different sample sizes were used for the simulations:

50,000, 100,000, and 400,000; however this decreased in the case-asymptomatic control analyses based upon the number of subthreshold observations that were excluded during the analyses. When simulating the data, we manipulated four factors: effect size (small = 0.025, moderate = 0.05, large = 0.1), minor allele frequency (0.01, 0.05, 0.1), population prevalence of the outcome (1%, 10%, 15%), and the prevalence of the subthreshold category (half the population prevalence of the cases, equal to the population prevalence, and double the population prevalence). The data were analyzed using probit or ordered probit regression models, as appropriate. To obtain consistent estimates, we simulated 1000 datasets for each combination of the manipulated factors, and the mean of the results for each combination of the parameters is presented.

### 2.2| Genome Wide Association Analyses

We used the three case-control coding schemes to recode the major depressive disorder (MDD) data from the white, European sample from the UK Biobank (Table 1) and conducted corresponding GWAS. Data used in GWAS were limited to unrelated individuals who had corresponding genetic data; however, total sample sizes are larger when considering all individuals in the UK Biobank. The case-control status was defined based on responses to self-report professional diagnoses, CIDI short-form diagnostic criteria, and ICD9/ICD10 diagnostic codes (supplemental methods) (Peters & Andrews, 1995; Wittchen, 1994). The ordinal phenotype included three distinct groups. Subjects were deemed cases (MDD = 2) if they met the full criteria for lifetime MDD. They were coded as subthreshold, or subsyndromal (MDD = 1) if they met some but not all the diagnostic criteria; for example, they had depression or anhedonia and three other symptoms but did not have the five required for a clinical diagnosis. Asymptomatic controls (MDD = 0) were subjects that did not meet any criteria for case or subthreshold or answered no to all screening questions for MDD, did not have a history of taking psychotropic medication, did not report partaking in activities to self-treat depression or anxiety, and did not report having sought professional help for depression. Finally, standard case-control analyses had the same case definition (MDD = 2), and controls included subthreshold individuals (MDD = 1) and asymptomatic individuals (MDD = 0). Additionally, individuals who self-reported a professional diagnosis of schizophrenia, bipolar disorder, autism spectrum disorder, or selected prefer not to answer were removed from the data prior to all analyses.

Table 1
Sample Sizes for Different MDD GWAS

| GWAS Model | # of Cases | # of Subthreshold | # of Controls | Total Sample Size |
|---|---|---|---|---|
| Standard Case-control | 37231 | - | 90363 | 127594 |
| Case-Asymptomatic control | 37231 | - | 60783 | 98014 |
| Ordinal | 37231 | 29580 | 60783 | 127594 |

All GWAS for the demonstration analyses in the UK Biobank were conducted in GW-SEM (Pritikin et al., 2021) due to its ability to conduct GWAS on ordinal outcomes. All GWAS included the first ten genetic principal components, age, and biological sex as covariates. Secondary analyses used summary statistics from the GWAS and included functional annotation and gene-wise analyses using FUMA and MAGMA (Watanabe et al., 2017) and used LD score regression (LDSC) to estimate the proportion of variance due to common variants ($h^2_{SNP}$).

## 3 Results

### 3.1 | Simulation Results

We conducted a 3 (fixed effect size = 0.025, 0.05, 0.1) by 3 (MAF = 0.01, 0.05, 0.1) by 3 (subthreshold prevalence = half population disease prevalence, equal to population disease prevalence, double population disease prevalence) simulation study for 3 sample sizes (50,000, 100,000, 400,000) and 3 disease prevalence (0.01, 0.1, 0.15). Because the results are extremely similar across conditions, Fig. 1 presents the results of the simulation study for the fixed effect size of 0.05 and MAF of 0.05 across all population prevalence, subthreshold prevalence, and sample sizes. The results for the other combinations of simulation study parameters are presented in Supplemental Figures. Consistent with previous research, the power to detect genetic association increases proportional to the magnitude of the genetic effect size, the MAF, and the population prevalence. Accordingly, we focus on the two novel components of our simulation study: 1) the definition of the case-control status and 2) the relative size of the subthreshold group.

We find that across each combination of manipulated factors, the ordinal model consistently has the highest power to detect genetic associations, and the standard case-control model consistently has the least power to detect genetic associations. Importantly, while the power differential between the ordinal and standard case-control outcomes increases proportional to the size of the subthreshold category, we also see power increases between these two specifications for the larger effect sizes, MAFs, and population prevalence.

Second, the power of the case-asymptomatic control reflected either the ordinal or standard case-control models depending on the size of the subthreshold category. At the lowest population prevalence, the case-control and case-asymptomatic control models have virtually the same power to detect genetic associations with minimal differences across the other factors. As the population prevalence increases, the power to detect genetic associations in the asymptomatic model increases faster than the standard case-control model to approach the power of the ordinal model.

### 3.2 | GWAS Results

#### 3.2.1 | Major Depressive Disorder

To extend the findings of the simulation study, we conducted a demonstration GWAS for major depressive disorder (MDD) using ordinal case-subthreshold-asymptomatic, case-asymptomatic control and standard case-control coding. The results of the MDD GWAS, presented in Fig. 2 identified three genome-wide significant loci across all three specifications on chromosomes 6, 7, and 22, respectively.

For the locus on chromosome 6, the ordinal and case-asymptomatic control models have the same lead SNP, rs3131113, and the ordinal model was more significant ($p = 8.96 \times 10^{-9}$) than the case-asymptomatic control ($p = 5.0 \times 10^{-8}$). The standard case-control model had a different lead SNP, rs3131115, which is in high linkage disequilibrium with the lead SNP from the other models and had similar significance levels to the case-asymptomatic control model ($p = 2.78 \times 10^{-8}$) (see Fig. 3). Interestingly, the lead SNP from the ordinal and case-asymptomatic control analysis does not reach genome-wide significance in the standard case-control model. Furthermore, it is important to note that the genome-wide significant locus on chromosome 6 falls within the Major Histocompatibility Complex (MHC) region, which is known for complicating the inference and interpretation of associated SNPs and genes due to the region's high levels of polymorphism and structural variation (Dilthey, 2021). Consistent with the simulation study, in the ordinal and case-asymptomatic control analyses, the significant variant in the locus on chromosome 7, rs3807866, was similarly significant ($p = 2.60 \times 10^{-9}$ and $p = 3.61 \times 10^{-9}$, respectively), but the standard case-control GWAS p-value was less significant ($p = 5.19 \times 10^{-8}$). A different pattern of results emerged for the significant variant on chromosome 22, rs143096365, which had similar levels of significance in the ordinal and standard case-control models ($p = 3.00 \times 10^{-10}$ and $p = 1.85 \times 10^{-10}$) and a higher significance in the case-asymptomatic control model ($p = 4.14 \times 10^{-11}$).

In contrast to the simulations, the lead variants in the case-asymptomatic control analyses on chromosome 22 were slightly more significant than those in the ordinal analyses, as seen in the qq-plot of the results (Fig. 4).

However, consistent with the simulations, the test statistics from the ordinal and case-asymptomatic control analyses in the suggestive significance range are more significant than the standard case-control model supporting increased power for these models.

To quantify the increase in statistical power in terms of sample size, we estimated the number of additional individuals in the standard case control analysis that would be required to obtain the same level of power as the ordinal model using non-centrality parameters (Verhulst, 2017). For the association on chromosome 6 (rs3131113), the standard case control model would have required 8,020 more people, and for the genome-wide significant SNP on chromosome 7 (rs3807866) the standard case control model would have needed 11,962 more people. Finally, the levels of power for the ordinal and standard case control models for the genome-wide significant SNP on chromosome 22 (rs143096365) were approximately the same, and no additional people would have been required in the standard case control model.

The results of the gene-based analyses conducted in MAGMA are presented in Fig. 5.

Analyses for all GWAS models identified multiple genome-wide significant gene-based associations. Specifically, five genes were significant for both the ordinal and case-asymptomatic control GWAS: TMEM106B on chromosome 7, which codes for a transmembrane protein that is involved in lysosome transport in neurons; a series of 3 significant genes that code for parts of histone complexes on chromosome 6 (HIST1H2AL, HIST1H2BN, HIST1H2BL); and DCC on chromosome 18, which encodes a netrin1 receptor and is involved in axon guidance required for neuronal cone growth. Interestingly, the case-asymptomatic control and ordinal GWAS also identified a significant gene on chromosome 3 (C3orf84) that is a protein coding open reading frame and a significant gene on chromosome 1 (ST6GALNAC3). Furthermore, in addition to the genes on chromosomes 1 and 3, the ordinal analysis identified an additional significant gene on chromosome 6 that codes for another part of the histone complex (HIST1H2AJ) and an additional significant gene on chromosome 18 (TCF4) that codes for a protein involved in regulating DNA expression.

The genome-wide significant SNP associations identified in the ordinal MDD GWAS correspond with existing analyses of MDD (Cai et al., 2020; Coleman, Peyrot, et al., 2020; Howard et al., 2018, 2019; Levey et al., 2021; Wray et al., 2018) and related traits (Levey et al., 2020; Purves et al., 2020). Genes identified in MAGMA also correspond to previous studies using an MDD or related phenotype. Specifically, TMEM106B has been previously associated with unipolar depression (Giannakopoulou et al., 2021; Howard et al., 2019; Wray et al., 2018), broad depression (Howard et al., 2018), neuroticism (Nagel et al., 2018), and lifetime anxiety disorder (Purves et al., 2020). C3orf84 has been associated with unipolar depression (Howard et al., 2019; Mitchell et al., 2022). ST6GALNAC3 has been associated with variations in electroencephalograms observed in multiple psychiatric disorders including schizophrenia, depression, and anxiety disorders (Hodgkinson et al., 2010), bipolar disorder (Swaminathan et al., 2015), and in gene by environment interactions for childhood maltreatment and different psychiatric phenotypes (Warrier et al., 2021). TCF4 has been associated with multiple psychiatric phenotypes including schizophrenia, bipolar disorder, depression, and post-traumatic stress disorder (Teixeira et al., 2021). Finally, DCC has been specifically associated with unipolar depression (Coleman, Gaspar, et al., 2020; Wray et al., 2018), neuroticism (Nagel et al., 2018), and bipolar disorder (Coleman, Gaspar, et al., 2020) and has also been implicated in the cross-disorder genetic architecture for numerous psychiatric disorders (Cross-Disorder Group of the Psychiatric Genomics Consortium, 2019) .

The phenotypic variance due to common genetic variants, or SNP heritability ($h^2_{SNP}$), was estimated for each of the GWAS summary statistics using linkage disequilibrium score regression (LDSC) (Table 2). While the $h^2_{SNP}$ estimates were broadly similar across the three analyses, the case-asymptomatic control analysis ($h^2_{SNP}$ = 0.10) had significantly higher heritability estimates than the ordinal analysis ($h^2_{SNP}$ = 0.08), which in turn had significantly higher heritability estimates than the standard case-control analysis ($h^2_{SNP}$ = 0.06). The magnitude and variability in the $h^2_{SNP}$ estimates in the current analyses reflect the heterogeneity in the heritability estimates for the existing MDD GWAS literature (Howard et al., 2018, 2019; Levey et al., 2021; Wray et al., 2018; Hyde et al., 2016). The $h^2_{SNP}$ for the case-asymptomatic control analysis should be interpreted with caution, as the exclusion of the subthreshold observations imply a complicated ascertainment strategy where the GWAS regression coefficients are likely overestimated which would inflate heritability estimates. Notably, while the $\lambda_{GC}$ values were greater than 1 for all of the analyses, the LDSC intercepts did not differ from 1, suggesting the results are consistent with an interpretation of polygenicity rather than genomic inflation for all of the coding schemes (Bulik-Sullivan et al., 2015).

Table 2
Heritability estimates for each GWAS

| Phenotype | $h^2_{SNP}$ (se) | LDSC Intercept | $\lambda_{GC}$ |
|---|---|---|---|
| Standard | 0.0595 (0.005) | 1.0061 | 1.15 |
| Asymptomatic | 0.0981 (0.0067) | 1.0002 | 1.18 |
| Ordinal | 0.079 (0.005) | 0.9968 | 1.19 |

## 4 Discussion

Our simulation study and empirical analysis of MDD demonstrate that the definition of the control group in GWAS increases the power to detect genetic associations. Specifically, the simulation study showed that an ordinal model, which includes an intermediate group of subthreshold individuals, consistently has more power to detect genetic associations relative to the standard case-control model. The enhanced performance is a function of capitalizing on richer phenotypic definitions than is possible in standard case-control models. In addition, the power of the case-asymptomatic model, where the subthreshold group is excluded, reflects either the ordinal or standard case-control models under different circumstances. For rare disorders, the power of the case-asymptomatic coding scheme is on par with the standard case-control methods, but as the population prevalence increases, the power to detect genetic associations increases relative to the standard case-control model.

Power differences across the three coding schemes are a direct reflection of the liability threshold model under which individuals with the highest genetic load for the disorder will have the highest liability (Neale, 2005; Verhulst & Neale, 2021). Controls, by extension, consist of individuals whose symptoms do not reach the diagnostic threshold. Depending on the diagnostic threshold, a large degree of variation may exist for both cases and controls (though we focus on the controls). Accordingly, by separating controls into subthreshold and asymptomatic groups by adding an additional threshold to the liability dimension, we increase the information in the analysis which improves statistical power (Manchia et al., 2013).

Figure 6 presents a schematic depiction of how the liability-threshold model affects statistical power for the three models. For simplicity, assume that the minor allele frequency, the population prevalence of the cases, and the genetic association with the liability dimension are constant across models. Now, if the subthreshold group is relatively small, the difference between the means (on the liability scale) of the standard control group and the asymptomatic group will be quite small. This would be typical of less prevalent disorders like schizophrenia. As the size of the subthreshold group increases, the asymptomatic group will become more extreme, and the difference between the means of the standard control group and the asymptomatic group will increase, thereby increasing the power to detect differences in both the ordinal and asymptomatic models relative to the standard case-control method. This would be typical of more common disorders like depression and anxiety where many individuals in the population experience subthreshold symptoms. Because the ordinal group retains the entire sample, statistical power will increase faster in the ordinal model due to inherent additional information. If the subthreshold group becomes too large, however, the power in the asymptomatic model will begin to decline from the reduction in sample size. Furthermore, results presented from case-asymptomatic control must be interpreted with caution as they may have inflated effect size estimates due to the more extreme differences between cases and controls, which would result in biases in post-GWAS estimates, like heritability (Nudel et al., 2020; Schork et al., 2019; Yap et al., 2018)

Our GWAS analysis of MDD generally supported the simulation study findings. The ordinal GWAS identified genome-wide significant associations in the same regions as the case-asymptomatic control and standard case-control GWAS. Consistent with the simulation results, for the vast majority of the loci analyzed, the ordinal results were slightly more significant than the case-asymptomatic control results which were more significant than the standard case-control results, as shown in the qq-plot (Fig. 3). In contrast with the simulation results which suggested the ordinal analysis would be the most powerful, some of the associations for the case-asymptomatic results were slightly more significant than the ordinal associations at those loci (particularly the associations on chromosome 22). While it is important not to overinterpret these minor differences, it is possible that nonlinearities (potentially resulting from dominance or epistasis) in the liability distribution could drive these unanticipated effects. As most GWAS current analyses are underpowered to detect dominance, we leave this possible explanation for future research.

The pattern of findings is consistent with the interpretation that our results reflect an increase in the statistical power to detect genetic associations rather than an increase in the false positive rate as we see virtually no inflation of the LDSC intercept (Table 2). Furthermore, as the locus zoom plots for chromosome 6 (Fig. 4) demonstrate, the general pattern of associations for all models is similar; however, the level of significance varies across models. The ordinal model has the highest level of significance, whereas the SNP is barely over the threshold in the case-asymptomatic control model. The locus on chromosome 6 is barely genome-wide significant in the standard case-control and case-asymptomatic control models but comfortably genome-wide significant in the ordinal model. Overall, even though the ordinal model only provides a modest power advantage, the impact of this advantage should not be understated.

Our results should be interpreted in light of several limitations. First, we interpret our results within the context of the liability threshold model assuming a unidimensional liability. If the liability is multidimensional, meaning that the liability of the subthreshold group does not fall exactly between the cases and asymptomatic controls, the pattern of results we present may not hold. For example, if a group of respondents report subthreshold symptomatology for MDD because they are taking an antidepressant, coding them as subthreshold would reduce statistical power. This emphasizes the importance of using lifetime disorders rather than recent symptoms as the outcome phenotype. Second, the phenotypic information necessary to adequately classify subthreshold cases may not be available in the data. In this situation, misclassification of subthreshold individuals as asymptomatic controls would also reduce the statistical power. Thus, it is essential that the analytical strategy is consistent with the data.

Oftentimes in case-control GWAS, controls are simply defined by the absence of the phenotype of interest. The current literature tends to focus on the strict definition of cases and places less emphasis on controls, which we argue is suboptimal as information and power are lost even in well-designed studies (Tsuang et al., 1993; Van der Sluis et al., 2010; Waszczuk et al., 2020). In our simulation study and demonstration analysis, we proposed an alternative operationalization of the control group definitions for GWAS analyses that can increase power to detect genetic associations. As such, asymptomatic models will benefit from these innovations.

### Declarations

## Author Contribution

J.H. and B.V. conceptualized the study. S.B. and B.V. wrote the main manuscript text. K.W. and S.B. conducted the analyses. S.B. prepared the figures. All authors reviewed the manuscript.

## Acknowledgements

This work was supported by NIH award R01MH113665.

#### Conflict of Interest Statement

The authors do not have any conflicts of interest to report.

## Data Availability Statement

Data was accessed through the UK Biobank application number 57923. Data can be accessed through the UK Biobank AMS

## Figures

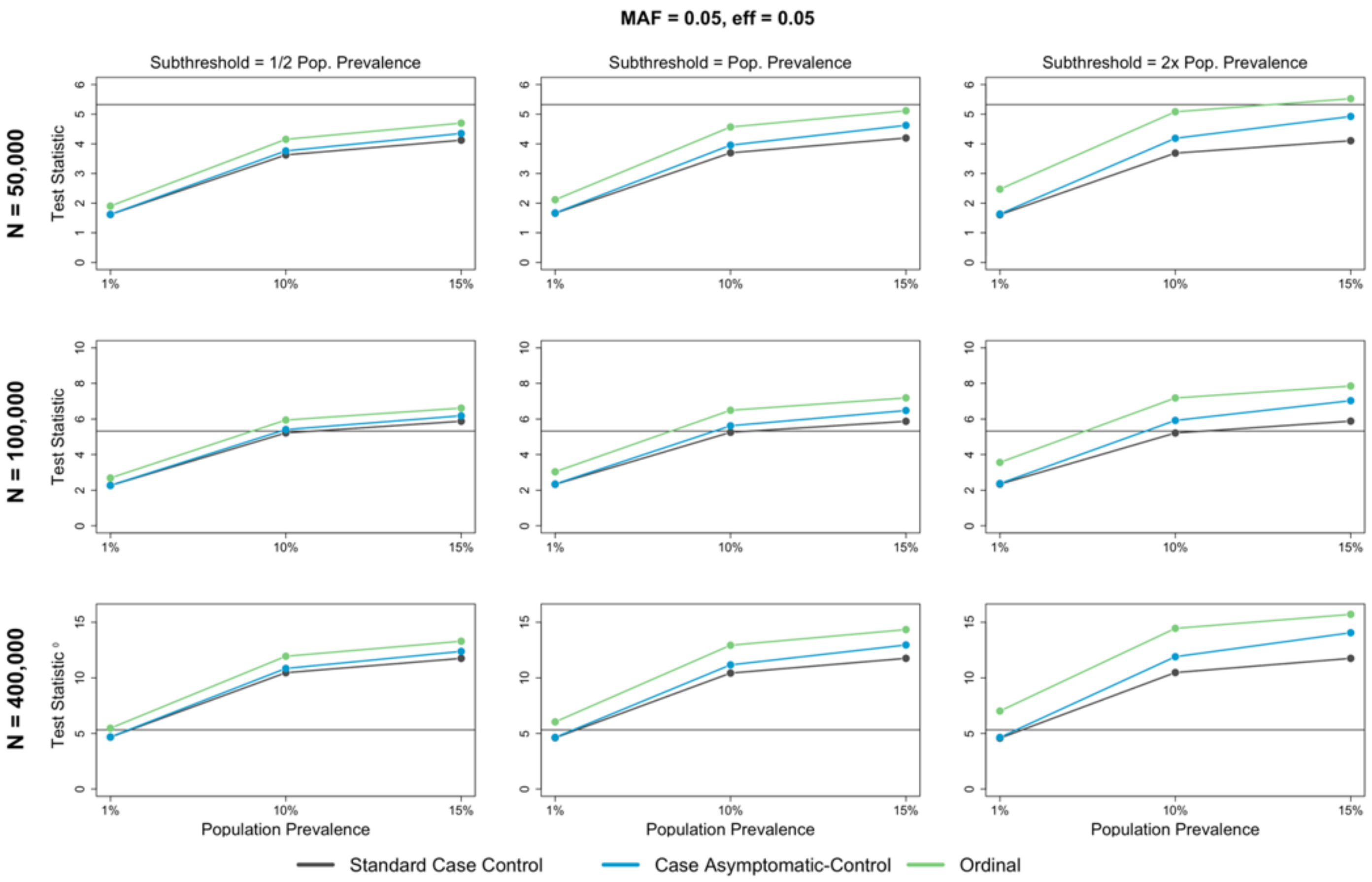

### Figure 1

Scatterplots of the simulation results at an effect size of 0.05 and a minor allele frequency of 0.05, where the average test statistic represents statistical power to detect associations, and the black line across all plots represents genome-wide significance

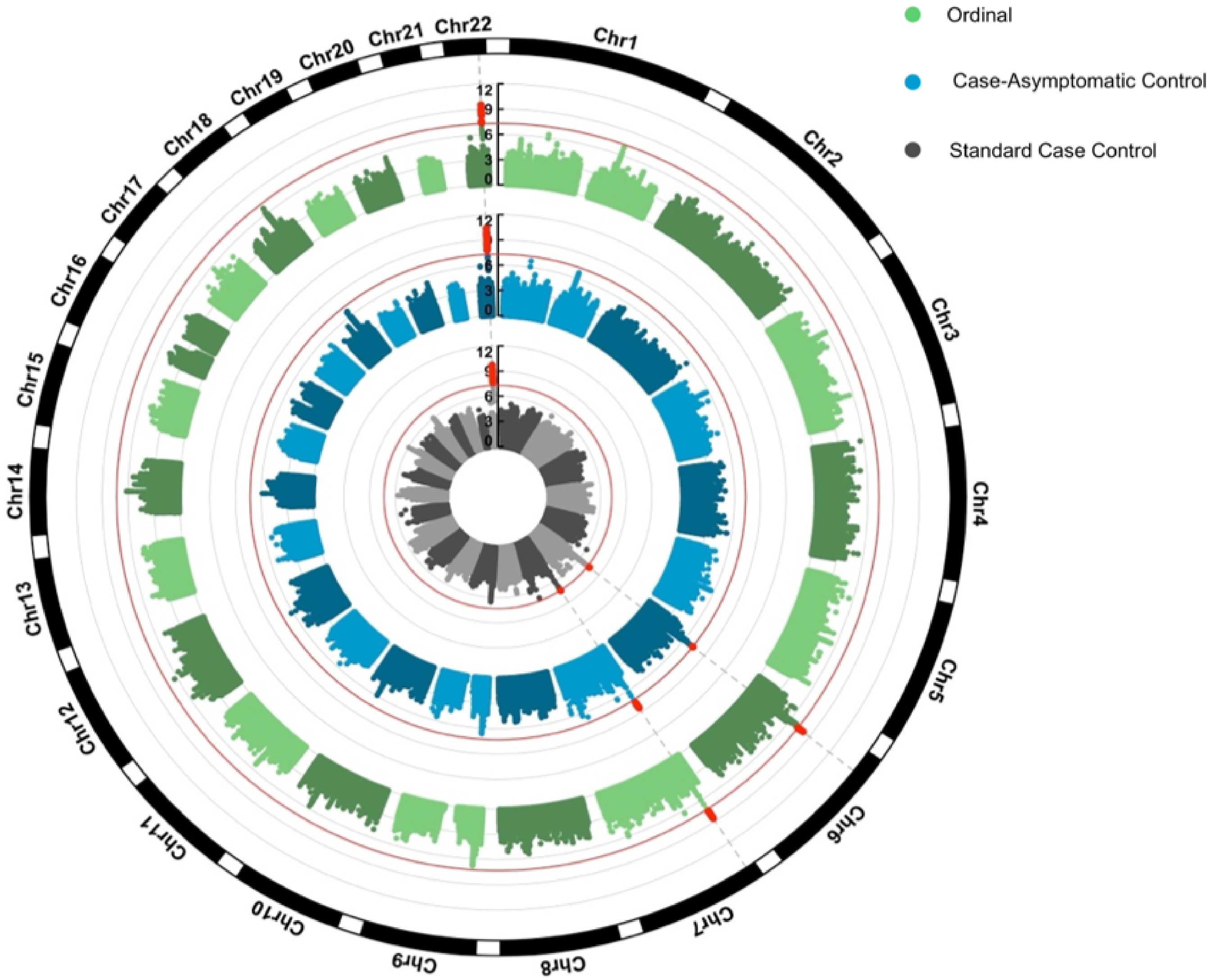

### Figure 2

Circular Manhattan plot of summary statistics from all MDD GWAS where the genome-wide significance line is in red. The ordinal model (outermost circle) contains three genome-wide significant hits denoted by red markers. The case-asymptomatic control model (middle circle) contains three genome-wide significant hits. The standard case-control model (innermost circle) contains three genome-wide significant hits. The level significance of the SNPs varies across the models

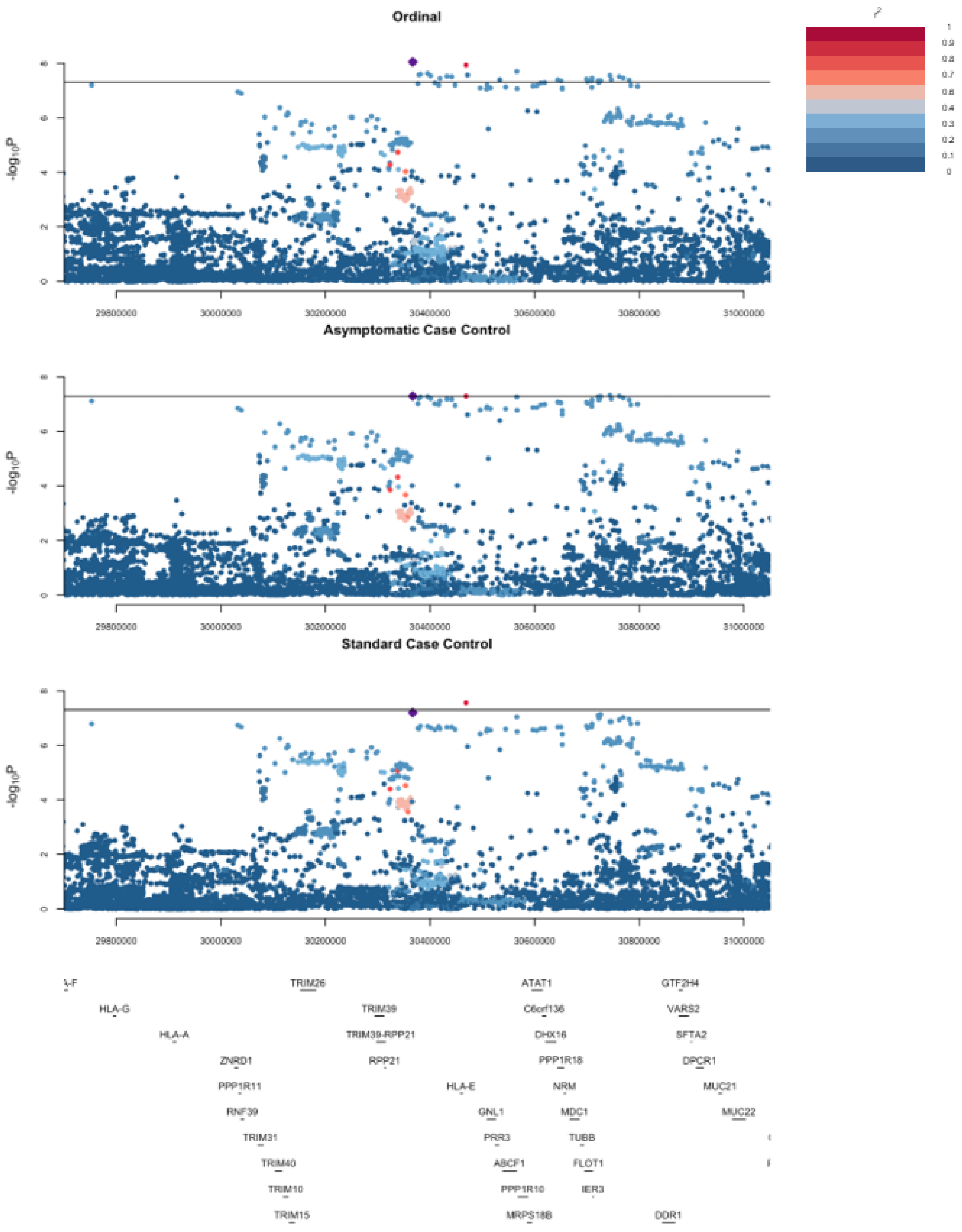

### Figure 3

Regional plots of the genome-wide significant locus on Chromosome 6 for the Ordinal, Asymptomatic Case-Control, and Standard Case-Control Models from the demonstration using MDD. The lead SNP from the ordinal and Asymptomatic Case-control models is denoted by the purple diamond. Protein coding genes in the region are displayed at the bottom of the plots

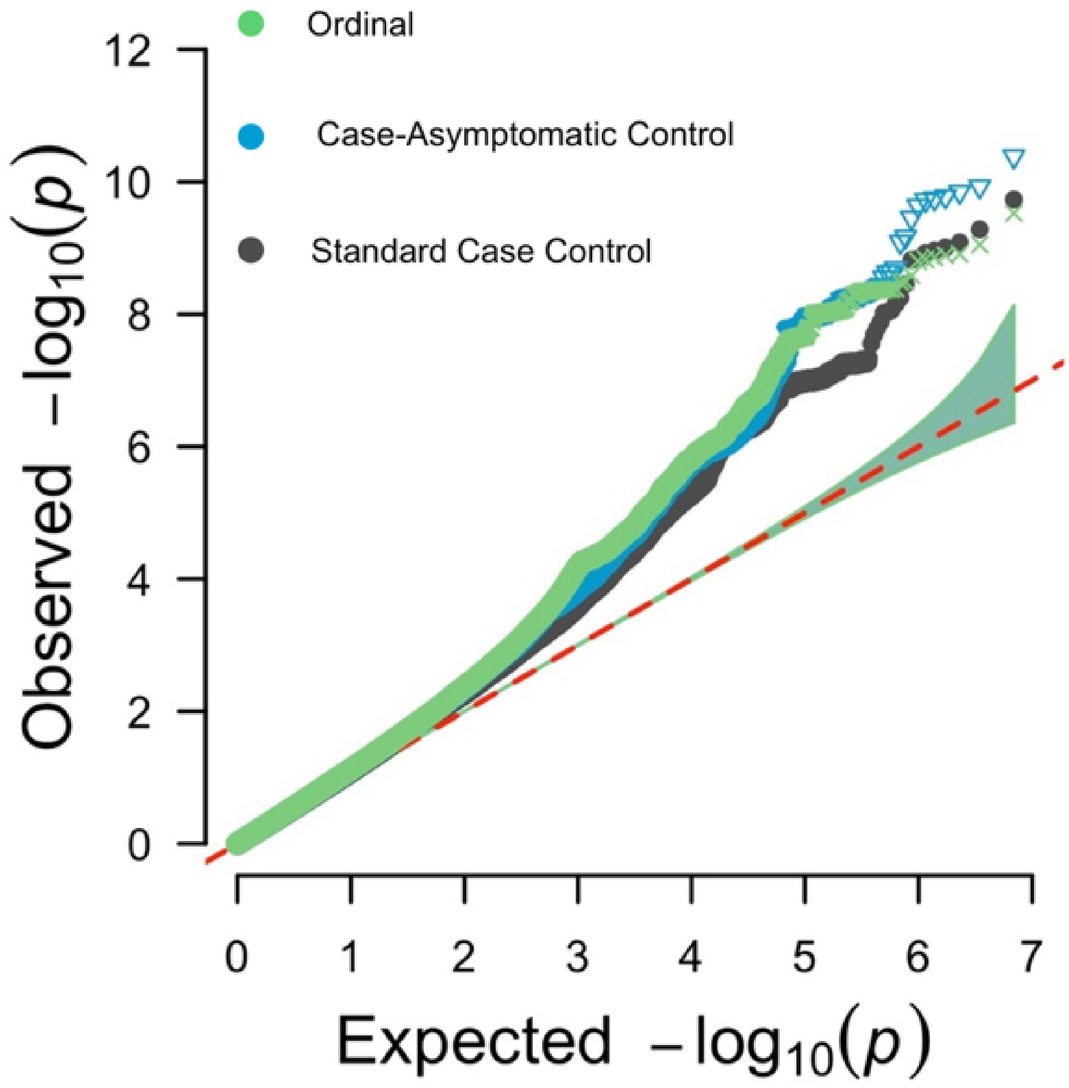

**Figure 4**

QQ-plot of the MDD GWAS results

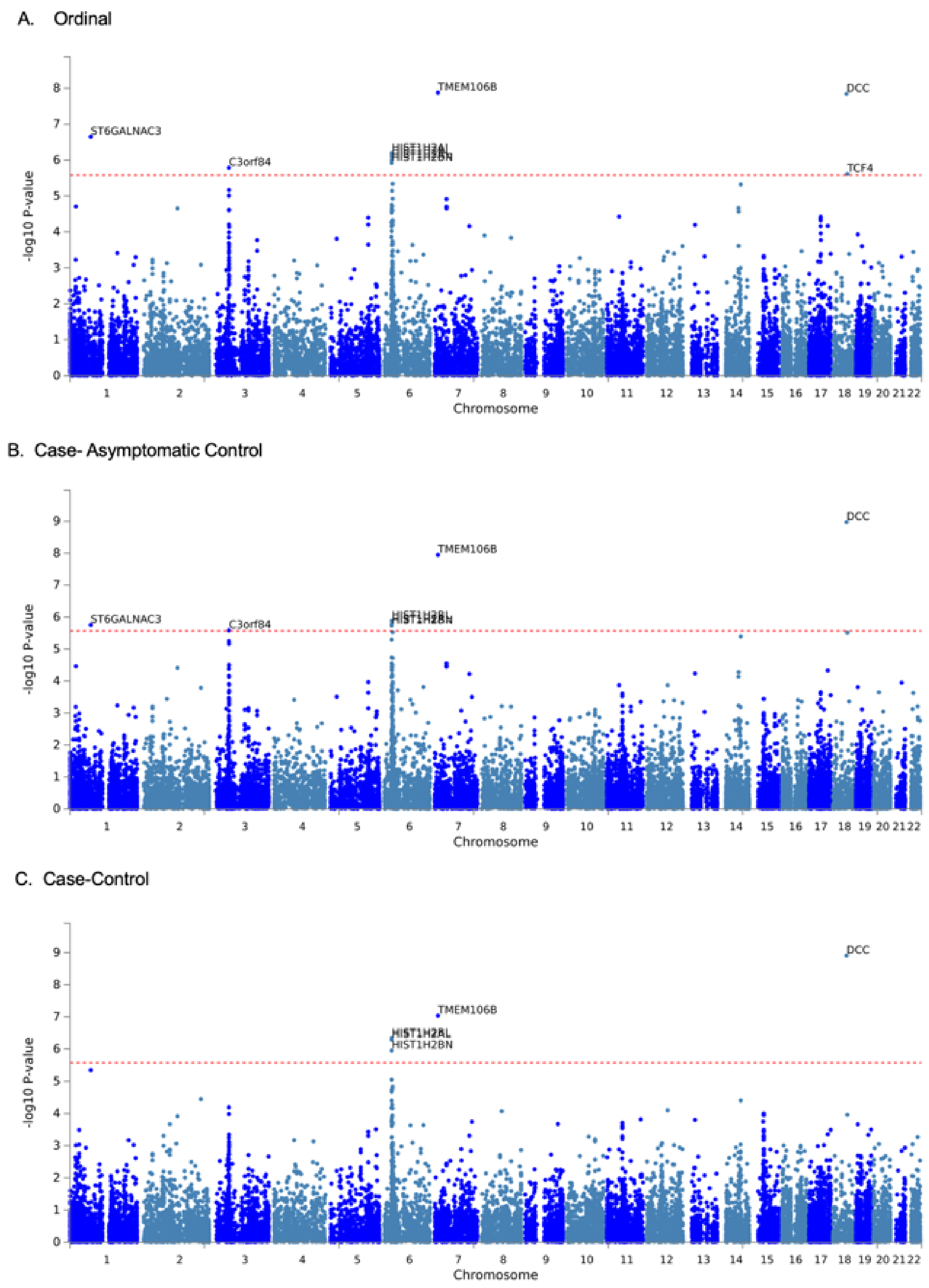

### Figure 5

Gene-based Manhattan plots from MAGMA. (A.) The Ordinal gene-based tests showed 9 genome-wide significant genes. (B.) The Asymptomatic Case-control gene-based tests showed 7 genome-wide significant genes. (C.) The Standard Case-control gene-based tests showed 5 genome-wide significant genes

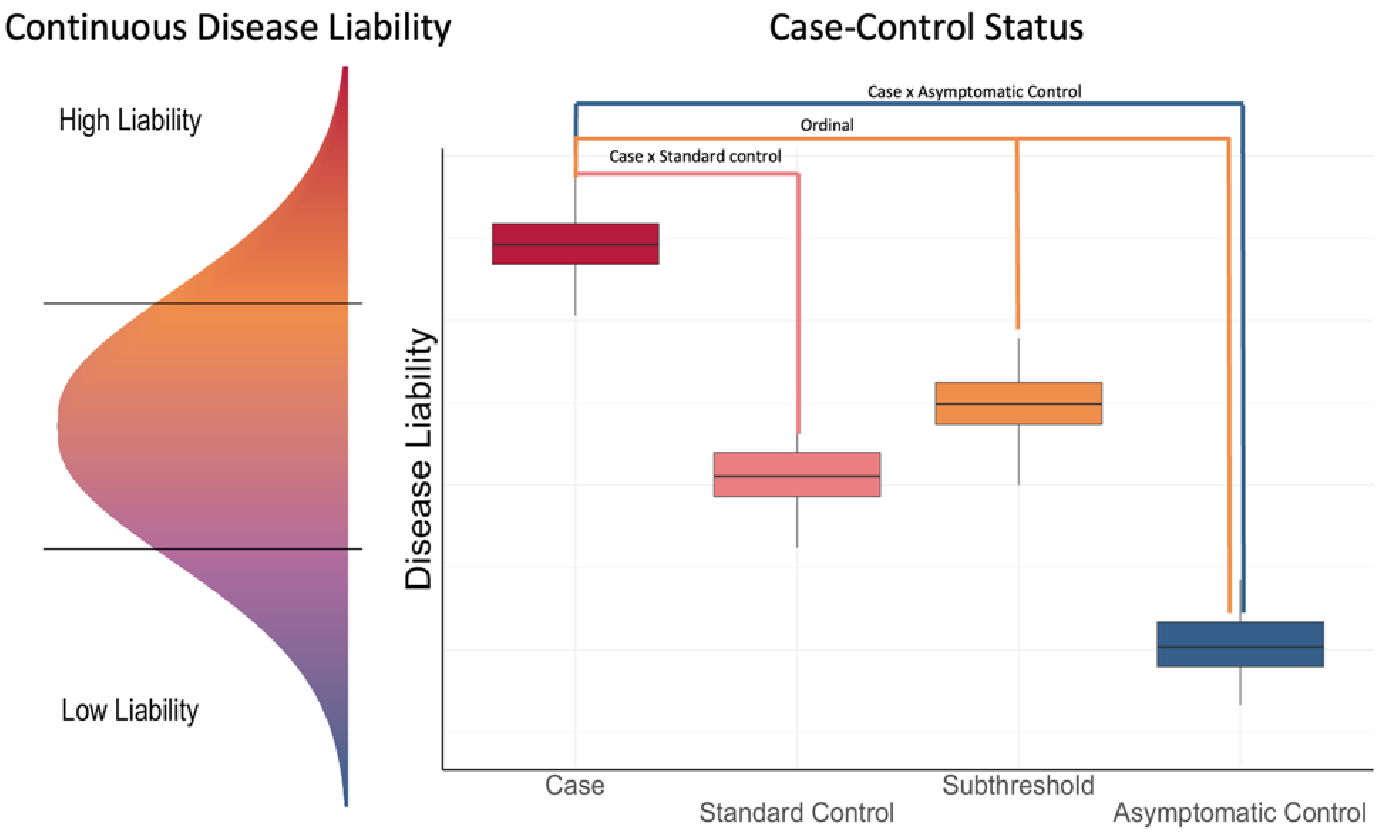

**Figure 6**

Theoretical Fig depicting the differences between categorical phenotypic groups and their place on a continuous disease liability scale. The largest difference is between Cases and Asymptomatic Controls (blue), and the smallest difference is between Cases and Standard Controls (pink). While multiple levels of differences are accounted for in an ordinal approach (orange)

## Supplementary Files

This is a list of supplementary files associated with this preprint. Click to download.

- SupplementalFigures.docx
- S
- SupplementalMethodsforUKBDemonstration.docx
